# The Validity of the Energy Expenditure Criteria Based on Open Source Code through two Inertial Sensors

**DOI:** 10.3390/s22072552

**Published:** 2022-03-26

**Authors:** Jaime Martín-Martín, Li Wang, Irene De-Torres, Adrian Escriche-Escuder, Manuel González-Sánchez, Antonio Muro-Culebras, Cristina Roldán-Jiménez, María Ruiz-Muñoz, Fermín Mayoral-Cleries, Attila Biró, Wen Tang, Borjanka Nikolova, Alfredo Salvatore, Antonio I. Cuesta-Vargas

**Affiliations:** 1 Biomedical Research Institute of Málaga (IBIMA), 29010 Málaga, Spain; jaimemartinmartin@uma.es (J.M.-M.); detorres.irene@gmail.com (I.D.-T.); adrianescriche@gmail.com (A.E.-E.); mgsa23@uma.es (M.G.-S.); amuro@uma.es (A.M.-C.); cristina.roldan005@gmail.com (C.R.-J.); marumu@uma.es (M.R.-M.); fermin.mayoral.sspa@juntadeandalucia.es (F.M.-C.); 2 Legal and Forensic Medicine Area, Department of Human Anatomy, Legal Medicine and History of Science, Faculty of Medicine, University of Málaga, 29071 Málaga, Spain; 3 Faculty of Media and Communication, Bournemouth University, Bournemouth BH12 5BB, UK; lwang@bournemouth.ac.uk; 4 Physical Medicine and Rehabilitation Unit, Regional Universitary Hospital of Málaga, 29010 Málaga, Spain; 5 Department of Physiotherapy, University of Málaga, 29071 Málaga, Spain; 6 Department of Nursing and Podiatry, University of Málaga, 29071 Málaga, Spain; 7 Mental Health Unit, Regional Universitary Hospital of Málaga, 29010 Málaga, Spain; 8 ITWare, 1117 Budapest, Hungary; attila.biro@itware.hu; 9 Faculty of Science and Technology, Bournemouth University, Bournemouth BH12 5BB, UK; wtang@bournemouth.ac.uk; 10 Arthaus, Production Trade and Service Company Arthaus Doo Import-Export Skopje, 1000 Skopje, North Macedonia; borjanka@arthaus.mk; 11 Sensor ID Snc., 86021 Boiano, Italy; alfredo.salvatore@sensorid.it; 12 School of Clinical Science, Faculty of Health Science, Queensland University Technology, Brisbane 400, Australia

**Keywords:** inertial sensors, energy expenditure, open source R, validation, walk, run, equations, assessment

## Abstract

Through this study, we developed and validated a system for energy expenditure calculation, which only requires low-cost inertial sensors and open source R software. Five healthy subjects ran at ten different speeds while their kinematic variables were recorded on the thigh and wrist. Two ActiGraph wireless inertial sensors and a low-cost Bluetooth-based inertial sensor (Lis2DH12), assembled by SensorID, were used. Ten energy expenditure equations were automatically calculated in a developed open source R software (our own creation). A correlation analysis was used to compare the results of the energy expenditure equations. A high interclass correlation coefficient of estimated energy expenditure on the thigh and wrist was observed with an Actigraph and Sensor ID accelerometer; the corrected Freedson equation showed the highest values, and the Santos-Lozano vector magnitude equation and Sasaki equation demonstrated the lowest one. Energy expenditure was compared between the wrist and thigh and showed low correlation values. Despite the positive results obtained, it was necessary to design specific equations for the estimation of energy expenditure measured with inertial sensors on the thigh. The use of the same formula equation in two different placements did not report a positive interclass correlation coefficient.

## 1. Introduction

The estimation of energy expenditure (EE) is fundamental to determine the level of physical activity of a person. The current society we live in has eminently sedentary behaviour, which can produce serious problems, and along with other unhealthy lifestyle habits, means a change in lifestyle is necessary [1,2]. Likewise, the use of behaviour modification techniques alone can produce an increase in physical activity [3]. Use of these, in conjunction with physical activity monitors and suggests an increase in physical activity. Despite the possible technical difficulties that may exist during the process, a motivation to be physically active is increased through self-monitoring and goal setting, among others [4]. It is, therefore, necessary to design valid systems for the estimation of EE that are accessible and reliable.

Energy expenditure is the most popular way to assess physical activity carried out by the subject throughout the day, as well as to record the prolonged periods of inactivity (sleeping or seated) [5]. In general, this consumption is recorded in the metabolic equivalents (METs) unit. METs is a direct adaptation of “mass-specific energy costs, computed by taking the energy costs (VO2 mL∙kg^−1^∙min^−1^) and dividing them by 3.5 mL∙kg^−1^∙min^−1^”, according to the Compendium of Physical Activities [6]. In other words, an indirect calorimetry can be used as an estimation instrument to determine the energy expenditure of different activities. However, the formula mentioned above cannot be applied directly to all subjects because it does not consider individual variables, such as age, weight, height, or gender. Thus, correction factors should be used [7]. 

Physical activities are categorised based on their intensity and consumption of EE. In this research, the categories are established as followings: ˂1.5 METs light intensity, ˂3 METs moderate intensity, ˂6 METs vigorous intensity, ˃6 METs very vigorous intensity [8]. Nowadays, equipped with low-cost inertial sensors, wearable devices (e.g., activity monitors) have become powerful [9], and they are capable of recording physical activities and estimating them by METs in a real-time manner. However, detailed procedure information is still missing in published studies, for using these low-cost devices. For example, the following details are still unclear to others: accelerometer brand, placement, duration of measurement, data processing, and interpretation [10], which makes it difficult to reproduce similar results. In addition, most of the manufacturers choose to design their own systems, especially for calculating calories consumed or energy expenditure, and there are no fair comparisons between these low-cost devices [11]. In other words, the reliability of wearable devices for measuring fitness-related indicators has been analysed, but the estimation accuracy of energy consumption is still inadequate [12]. Besides, machine learning systems have been used to calculate energy expenditure. However, it is not reproducible in daily life as the high predictive precision of the models is calibrated under strict laboratory conditions [13]. Therefore, it is necessary to apply nonlinear models to obtain a reliable and easier EE estimation for daily practise [14,15]. 

Previous studies showed the measurement of energy consumption in different devices (Apple Watch 2, Samsung Gear S3, Jawbone Up3, Fitbit Surge, Huawei Talk Band B3, and Xiaomi Mi Band 2) was inadequate, and their mean absolute percentage errors reached 0.44. This shows the low reliability of devices, such as the Fitbit or the Jawbone, to estimate energy expenditure [16]. In addition, measurement accuracies varied between subjects individually, from 0.10 to 0.50, depending on the activity performed [8]. Previous studies have also demonstrated the existence of significant differences in energy expenditure or physical activity when climbing or descending stairs and even walking at low speeds when comparing specific devices designed for this purpose [17]. In this sense, the estimation of energy expenditure with smartwatches or similar devices in comparison with a gold standard (gas analysis data from indirect calorimetry or oxygen volume consumption) have also shown significant differences. These differences are smaller when the outcome variable heart rate is taken as a reference [18]. Likewise, specific device designs based on inertial sensors for energy expenditure estimation compared to gas analysis can obtain better results than those obtained by a conventional smartwatch or an activity-specific smartwatch [19]. Despite the improvement in the results based on specific systems, there are still differences in the estimation of energy expenditure that could be reduced.

Two of the most used inertial sensors for estimating energy expenditure are ActiGraph [20] and Genea [21], which are regarded as the gold standard when compared to new devices appearing in the market [22,23,24]. However, these devices have enclosed software and signatures with multiple modes for energy expenditure calculation, which remains unclear how these devices calculate the calory consumption using inertial sensors [25]. Meanwhile, their high purchasing cost is not affordable for many clinical applications, such as physical activity promotion, breaking sedentary behaviour, and technical use for weight loss [26]. Therefore, this study aims to develop an open source system for the calculation of energy expenditure, which is based on low-cost inertial sensors Lis2DH12 (STMicroelectronics, Gienbra, Suiza) and ActiGraph GT9X Link (ActiGraph LLC, Pensacola, FL, USA).

## 2. Materials and Methods

### 2.1. Design

An analytical cross-sectional study using three inertial measurement units (IMU) or sensors used to record physical activities was conducted to design an open source R-code for estimating energy expenditure. Five healthy adults were recruited to perform twice a set of ten physical activities. The informed consent was approved accordingly. 

Three IMUs per participant were used for the present study—two Actigraph GT9X Links [27] and one SensorID [28]—following two configurations. First configuration: each participant was equipped with an Actigraph with a band and a SensorID in the wrist with a double tape band and another Actigraph sensor placed on the thigh with a band. Second configuration: each participant was equipped with an Actigraph with a band and a SensorID on the thigh with a double tape band and another Actigraph sensor placed in the wrist with a band. 

A set of ten physical activities on a treadmill were conducted in each configuration. The set of activities were walk or running at 10 different speeds: 1.4 km/h, 2.9 km/h, 4.3 km/h, 5 km/h, 6 km/h, 6.5 km/h, 7 km/h, 8 km/h, 9 km/h and 10 km/h, respectively. Participants performed each physical activity on the treadmill for 3 min, and no data was recorded in the first one minute (to ignore some unstable activity pattern); the next two minutes were recorded.

### 2.2. Devices

The ActiGraph GT9X Link [27] sensor is equipped with its own software and it was designed to estimate EE. It can be programmed to measure each activity, which makes it possible to be automatically activated for recording accelerometer data in a specific period. The sampling frequency of the device is 30–100 Hz with a dynamic range of measure ±8 g, and its data storage is 4 GB [27]. In the present study, it was configured from the manufacturer’s software to extract the RAWdata format from the accelerometer (x-, y-, z-axes). This sensor is widely used for the estimation of physical activities [10].

The IMU sensor of the SensorID uses the Lis2DH12 [29] chipset model and Bluetooth 5.0 for wireless connection, and the accelerometers of activities can only be recorded in a real-time manner. The device has a measuring range of ±16 g and a range of sampling frequency 1–1620 Hz. Additionally, the x-, y-, and z-axes data from the accelerometer were obtained.

The sampling rate of both ActiGraph sensors and the IMU sensor from SensorID was 30Hz in all experiments. Each participant completed the sets of physical activities.

### 2.3. R-Code Development

An R-Code was developed in order to estimate EE based on x-, y-, z-axes data obtained from the sensors. The average activity count value (counts per minute) of each activity for EE computation is calculated through the sum of activity count values (counts per second) divided by two (each activity lasts two minutes). For Crouter et al.’s equation [30], the average activity count value (counts per 10 s) is calculated by the sum of activity count values (counts per second) divided by 12 (2 min is equal to 12 multiply 10 s). The VM is calculated by the square root of the square of the three axes of data. For Santos-Lozano et al.’s equations [20], we choose the equations designed for all age groups. All the programming codes designed for reading the accelerometer data, EE computation and EE correction are written in R language by two of our researchers (J.M.-M. and L.W.) (available code: https://doi.org/10.6084/m9.figshare.12080730; accessed on 30 April 2021), and a public library called “activityCounts” is used to calculate the activity counts (counts per second) [31].

### 2.4. Data Analysis

In our experiments, five participants wearing three sensors completed two configurations of a set of ten physical activities, and each activity lasted two minutes. Therefore, we have 300 records of activity data (5 participants × 3 sensors × 2 configuration × 10 activities) and 600 min of data in total. To obtain a more precise estimation of EE, descriptive and anthropometric variables of the sample were obtained (age, body mass, height, and gender). The estimation of EE by equations usually does not concern the individual variations, such as age and body mass, which leads to an inaccurate predicted energy cost of activities within a specific individual [6]. We further corrected the EE values by considering the variation of each participant (Table 1).

After the data collection, a data cleaning procedure proceeded. The incorrect activity data due to initialization errors and device malfunction were removed during this procedure. We removed four invalid monitor data (removed t2221, t221, t2130, and t3127), which finally led to 296 valid recorded activities. To select the appropriate predictions of EE, we reviewed up-to-date EE estimation methods using accelerometer data for adults in order to best match the physical activities conducted in this work. After the literature review, five different equations were chosen for calculating EE (in METs) which were Freedson et al. [32], Crouter et al. [30], Santos-Lozano et al. [20], the equations of the vertical axis (VT), and vector magnitude (VM) and Sasaki et al. [33]. The equations used in the present studies were validated in the right hip [20,30,32,33].

### 2.5. Statistical Analysis

To assess the reliability of the EE estimation between the two devices, intraclass correlation analysis elements (ICC) were performed: alpha mixed pattern of two factors. The statistical analysis employs the covariances among the items. A high intraclass correlation coefficient (ICC) close to 1 indicates high similarity between values from the same group. ICC is a reliability index that reflects both degrees of correlation and agreement between measurements [34].

## 3. Results

An interclass correlation analysis was performed to compare the calculated EE, which was based on the accelerometer data obtained by the two inertial sensors: SensorID (Lis2DH12) and ActiGraph. Ten different equations were applied to calculate the EE for different speeds of activities like walking and running. In six out of ten equations in our experiments, no significant differences were observed in the comparisons of EE estimated with the inertial sensors in the same placements (see Table 2 and Table 3).

The average correlation index between the standardised elements of energy consumption on the wrist was 0.946 (Table 2). The speeds obtained for the best interclass correlation index (ICC) on the wrist were 2.9 km/h and 4.3 km/h. The speed of 6 km/h was the one that obtained the lowest standardised interelement correlation index on the wrist. The calculation of the corrected EE of Freedson et al. 1998 [32] and Santos-Lozano et al. VT 2013 [20] obtained the best correlation rates between inertial sensors. The EE equations of Sasaki et al. 2011 [33] and corrected Santos-Lozano et al. VT 2013 [35] obtained the lowest correlation rates in our system, ICC:0.111, and ICC:0.306, respectively (see Table 2).

The ICC for the EE calculated on the thigh is shown in Table 3. The average ICC for the standardised elements of different speeds was 0.935. The speeds that offered the best rates were 2.9 km/h and 4.3 km/h. The corrected EE equations of Crouter et al. 2010 [30] and Freedson et al. 1998 [32] (0.982 and 0.979, respectively) were the best correlation results when placing the inertial sensor (SensorID) on the thigh. The lowest correlation rates among elements were those obtained by the corrected equations of Santos-Lozano et al. VM 2013 [20] (ICC:0.022) and Sasaki et al. 2011 [24] (ICC:0.044). The speed with the lowest correlation index among standardised elements on the thigh position was 7 km/h (see Table 3). Significant differences observed in the EE comparison were the wrist and thigh with the ActiGraph sensor (see Table 4 for details).

## 4. Discussion

The ICC of six EE equations (ten in total) shows similar results when applied to the accelerometer data from two different inertial sensors. Therefore, both sensors are valid for the EE estimation. The processing and analysis of the data in R software proved to be an automated and efficient calculation method. Based on the results obtained, the reliability of low-cost sensors (Lis2DH12) is well recognised when compared to a high-performance sensor (like ActiGraph).

The results of the interclass correlation interval on the wrist (Table 2) and thigh (Table 3) were consistent at most of the activity speeds, but it was slightly better on the wrist. The estimation of EE at a low-speed (2.3 km/h and 4.3 km/h) had a higher interelement correlation index than higher speeds (9 km/h and 10 km/h). The results suggested that it is not possible to use the same energy expenditure equation for the wrist and thigh (Table 4). To avoid the possible underestimation and reflect reliable energy expenditure calculation, we corrected the EE equations by considering the individual variables, such as gender, body mass index and age, for all participants in our experiments (Corrected METs—Compendium of Physical Activities).

Before interpreting the results of this study, the following aspects should be considered. The results in relation to ICC and the estimation of EE should be taken with caution because of two factors. The estimated results of energy expenditure applying the different equations were not compared with a VO2 indirect calorimetry system due to the lack of necessary equipment; VO2 systems for data collection are expensive and therefore difficult to use in daily life for the calculation of energy expenditure, making it necessary to identify alternative systems [36]. The sample size of this study was five participants, which is small. However, each participant walked on the treadmill twice at all selected speeds. This involves a large amount of data. Hence, the established sample size defines a confidence level of 80% and a margin error of 20%.

In the present study, we used EE equations and their corresponding corrected versions. For example, we named the corrected version of “Freedson et al. 1998” as the “corrected Freedson et al. 1998” [32]. Freedson et al.’s equation obtained the best correlation on the wrist with and without correction among five different EE equations. However, on the thigh, the best equation with and without correction was Crouter et al. 2010 [30]. It is important to consider the usage of differential equations and their reliability in any system designed for estimating energy expenditure [9,10,35]. Moreover, standard EE systems should still be used when the energy cost of physical activities is computed for population sets [6,7].

Previous studies have analysed EE based on equations considering VM, body weight and the heart rate reserve of individuals in two different allocations. No significant differences were observed in the variables weight, dead height or body mass index among the participants in the present study. As shown in Table 1, gender and body mass index are considered in the Crouter et al. [30] and Santos-Lozano et al. [20] equations. In all the corrected equations, age is considered, in addition to the aforementioned variables. An earlier study [25] shows that a significantly improved accuracy of estimated EE is observed when the Freedson et al. equation considers individual variables, such as vector magnitude and body weight. However, we did not notice such a significant difference in ICC in our experiments between the traditional Freedon et al. equation and the corrected equation (adapted to vector magnitude and body weight). In contrast, the results of the Freedson et al. equation from the wrist and thigh showed a strong ICC (wrist: 0.945; thigh: 0.972). Results of greater consistency were obtained when the corrected version of the EE equation was applied (ICC wrist: 0.960; thigh: 0.979). Moreover, the energy expenditure data estimated by an inertial sensor and an EE equation should still be taken with caution, as the predicted EE could be significantly underestimated compared to the actual EE in different population groups. Any underestimation could increase along with the increasing intensity of the activities; multiple-stage analysis regression models revealed that age and weight were related to actual EE in both the older and younger groups [37]. Meanwhile, similar studies have shown that the corrected formula translated to a better energy expenditure prediction by adding a heart rate reserve [38,39].

The heart rate parameters with a wrist accelerometer improved energy expenditure estimation significantly [18,40]. Related studies have compared two inertial sensors on EE estimations using the same equations as in our study [41]. They demonstrated that outpatient activities (walking on 3.2 km/h, 4.2 km/h, 6.0 km/h, and jogging at 8.4 km/h) have the best agreement between the Active Style Pro sensor and ActiGraph, including any four EE equations used in the ActiGraph software. In our study, these four same equations and speeds of similar gait were also used, and we observed positive correlations when measuring with two different inertial sensors (see Table 2 and Table 3). The inertial sensors placed on the hip are more likely to calculate accurate EE when participants are doing physical activities that involve mobility of the lower limbs (e.g., household, stairs, walking, and running) and the upper limbs (e.g., laundry, window washing, dusting, dishes and sweeping) [38,42]. However, a better overall prediction of EE is obtained when the inertial sensors are placed on the wrist in our study [42]. However, Slade et al. have used systems based on two inertial sensors (shank and thigh) for the estimation of energy expenditure, which produces a big improvement. Even using a single sensor on the thigh can significantly reduce error in the estimation of energy expenditure obtained by the smartwatch [19].

Besides, we also observed that the results obtained from the wrist and thigh cannot be compared fairly due to the interclass relation coefficient’s weakness (see Table 4). Similar to this, recent studies have demonstrated the need to design specific energy expenditure equations when measured on the thigh [43]. In this sense, according to the results obtained in the present study, the Lis2DH12 sensor compared with the ActiGraph showed a high ICC in the calculation of energy expenditure used in both the thigh and the wrist in six of the equations used (Table 3 and Table 4). In this regard, it is important to consider the evolution of the inertial sensor itself, which is improving in technology and increasing in recording accuracy. For this reason, it is difficult to compare the use of two inertial sensors for the estimation of energy expenditure if they are not the same and if there is no third reliable measurement for the estimation of energy expenditure.

The proposed EE calculation system is developed in an open source R software, which is under a general public license [44], and this licence guarantees the freedom for all users to share and change it. In addition, the advantages of our system are that we only need a CSV file as input, which records the raw accelerometer data (including the time, accelerometer x-axis, accelerometer y-axis, accelerometer z-axis, and the vector of magnitude), and the calculation is a numerical computation which needs low computing resources. Thus, the system is suitable for low-cost or high-cost inertial sensors, and it can be easily further implemented in smart devices, and is independent of specific software. The users can modify the provided EE equations or add new EE equations freely.

The results obtained in this study demonstrate the feasibility of using different equations to estimate energy expenditure in an open source system. This system could be used by any manufacturer able to implement it in their devices and allow the development of third-party applications based on these systems. Devices, such as the Jawbone Up3 (Jowbone, San Francisco, CA, USA) and Fitbit Surge (Fitbit, San Francisco, CA, USA), showed positive correlations greater than 0.8 when using step counting or accelerometer steps; however, energy expenditure was underestimated compared to indirect and direct calorimetry [16]. Furthermore, there is no doubt that a system based on biomechanical analysis is more reliable for the estimation of energy expenditure, with an error under 13%, which is very far from the 44% obtained by the smartwatch [19]. However, the use of such a system on a daily basis could be a limitation for the end-user to carry multiple recording devices instead of just one. In this way, a machine learning system could also be used to estimate the EE; using nonlinear models based on dynamic recognition allows for a better functioning of these systems [13]. These systems can increase the accuracy of EE recognition by up to 7% [45]. However, it is necessary to first validate the sensor data [46].

Our future research will apply specific energy expenditure equations to the thigh, for example, recent EE equations designed for daily living, sports activities and machine learning systems. This would produce a more precise system of energy expenditure calculation for a greater number of activities. This would be essential to compare the energy expenditure obtained by equations with the VO2 via indirect calorimetry [19,47]. The applications of the proposed method can be related to any EE estimation for physical activities, such as physical activity promotion for walking/running, clinical applications, like sedentary behaviour change for health care, and technical use for losing weight. We noticed that the proposed EE calculation system is capable of running automat, and only requires low-cost inertial sensors and limited computational resources, thus we could develop a completely autonomous software that is easily implanted into smart devices with inertial sensors on the market.

The present study has some limitations. For example, this study cannot determine which one of the two inertial sensors (Lis2DH12 or ActiGraph) or which one of the equations is better for the EE estimation because an indirect calorimeter or doubly labelled water method was not used as a validation site. The present study has a limited sample of participants, which could produce a type 2 error: hypotheses null not rejected. However, ten different speeds have been tested, and two positions and ten different energy expenditure calculation formulas generated an extensive database. The present study did not use the heart rate reserve; however, we used two allocations for the sensors. These placements are more comfortable and easier to record physical activities. In addition, this study shows that low-cost accelerometers can be used to design a new computational EE calculation system, which is written in open source R software for energy expenditure research. Furthermore, this system could replace standard measurements, such as the ActiGraph software.

## 5. Conclusions

In our study, two inertial sensors (SensorID and ActiGraph) were used to calculate the EE for different speeds of physical activities, such as walking and running, and the way we estimated EE was by using an automated system written in open source R software. The conclusion of this study is that not all formulas for EE estimations provide the same reliability when using different inertial sensors. However, we noticed that similar energy expenditure results were produced by the EE equations of Freedson et al., Crouter et al., Santos-Lozano et al., and their corrected versions, which indicates that they can be used by two different inertial sensors. Based on the results obtained, equations like Freedson et al. and Crouter et al. should not be used on the wrist and thigh at the same time as they show negative correlations in our experiments. The proposed EE calculation system can be easily further improved to be a more precise system for a wide range of applications, such as physical activity promotion and daily behaviour change. Moreover, its nature of open source software, automation and low requirements of computation resources makes it suitable to be implanted into smart devices with inertial sensors.

## Figures and Tables

**Table 1:** Equations applied to calculate energy expenditure.

Freedson et al. 1998 [32]	$EE=1.439008+7.95e^{-4}\times AC_{VT}$	
Crouter et al. 2010 [30]	Walking/Running	$EE=2.294275\times \exp\left(8.4679e^{-5}\right)\times AC_{VT}^{\prime }$
Santos-Lozano et al. VT 2013 [20]	Adults	$EE=3.4002+5.3e^{-4}\times AC_{VT}-5.564e^{-2}\times BM+1.2789\times Gender$
Santos-Lozano et al. VM 2013 [20]	Adults	$EE=2.8323+5.4e^{-4}\times AC_{VM}-5.912e^{-2}\times BM+1.4410\times Gender$
Sasaki et al. 2011 [33]	$EE=8.63e^{-4}\times AC_{VM}+0.668876$	
Correction_METs	Female	HBequation=655.0955+1.8496×Height×100+9.5634×BM−4.6756×Age
	Male	HBequation=66.4730+5.0033×Height×100+13.7516×BM−6.7550×Age
	Corrected	$Corrected\_EE=other\;methods\;\left(METs\right)\times \frac{3.5}{HBequation\times \left(\frac{1000}{1440\times 5\times BM}\right)}$
VT: vertical axis; VM: vector of magnitude; $AC_{VT}$: activity counts on vertical axis, counts per min; $AC_{VT}^{\prime }$: activity counts on vertical axis, counts per 10 s; This is a special $AC_{VT}$ for Crouter et al. 2010; $AC_{VM}$: activity counts on vector of magnitude (three axes), counts per min; BM: body mass (kg); Height: cm; Age: year; Gender: 1: female, 2: male.		

**Table 2:** ICC results for energy expenditure computed from two inertial sensors (ActiGraph and Sensor ID) on the wrist.

Speed	Crombach’s Standardised Items	Energy Expenditure Calculation System									
		Freedson et al. 1998 [32]	Crouter et al. 2010 [30]	Santos-Lozano et al. VT 2013 [20]	Santos-Lozano et al. VM 2013 [20]	Sasaki et al. 2011 [33]	Corrected Freedson et al. 1998 [32]	Corrected Crouter et al. 2010 [30]	Corrected Santos-Lozano et al. VT 2013 [20]	Corrected Santos-Lozano et al. VM 2013 [20]	Corrected Sasaki et al. 2011 [33]
1.4 km/h	0.918	0.997	0.723	0.997	0.928	−0.331	0.997	0.651	0.997	0.904	0.457
2.9 km/h	0.992	0.992	0.941	0.994	0.928	0.979	0.993	0.800	0.994	0.958	0.926
4.3km/h	0.982	0.935	0.835	0.951	0.957	0.929	0.932	0.932	0.950	0.957	0.929
5 km/h	0.957	0.956	0.956	0.983	0.915	0.744	0.985	0.991	0.983	0.899	0.875
6 km/h	0.850	0.951	0.958	0.946	0.518	−0.005	0.957	0.968	0.956	0.110	−0.310
6.5 km/h	0.943	0.957	0.944	0.961	0.146	0.010	0.957	0.948	0.963	−0.119	−0.019
7 km/h	0.955	0.941	0.931	0.936	−0.359	−0.157	0.962	0.949	0.960	−0.357	−0.015
8 km/h	0.961	0.865	0.879	0.846	−0.151	0.015	0.871	0.861	0.841	−0.138	0.177
9 km/h	0.940	0.989	0.989	0.989	−0.613	−0.709	0.991	0.991	0.989	−0.301	0.032
10 km/h	0.961	0.865	0.819	0.858	−0.366	−0.361	0.959	0.897	0.933	0.148	0.225
MEAN	0.946	0.945	0.898	0.946	0.391	0.111	0.960	0.899	0.957	0.306	0.328

**Table 3:** ICC results for energy expenditure computed from two inertial sensors (ActiGraph and Sensor ID) on the thigh.

Speed	Crombach’s Standardised Items	Energy Expenditure Calculation System									
		Freedson et al. 1998 [32]	Crouter et al. 2010 [30]	Santos-Lozano et al. VT 2013 [20]	Santos-Lozano et al. VM 2013 [20]	Sasaki et al. 2011 [33]	Corrected Freedson et al. 1998 [32]	Corrected Crouter et al. 2010 [30]	Corrected Santos-Lozano et al. VT 2013 [20]	Corrected Santos-Lozano et al. VM 2013 [20]	Corrected Sasaki et al. 2011 [33]
1.4 km/h	0.887	0.990	0.987	0.997	0.804	−0.689	0.982	0.999	0.995	−0.007	−0.260
2.9 km/h	0.993	0.989	0.993	0.994	0.915	0.879	0.989	0.994	0.994	0.913	0.881
4.3 km/h	0.993	0.994	0.995	0.993	0.721	0.541	0.995	0.996	0.994	0.726	0.608
5 km/h	0.974	0.986	0.990	0.981	0.195	−0.176	0.989	0.991	0.987	−0.035	−0.279
6 km/h	0.953	0.950	0.955	0.949	−0.198	−0.361	0.968	0.968	0.964	−0.607	−0.544
6.5 km/h	0.954	0.996	0.996	0.989	−0.327	−0.441	0.989	0.987	0.984	−0.702	−0.555
7 km/h	0.716	0.916	0.930	0.897	0.139	−0.071	0.952	0.953	0.947	−0.763	−0.660
8km/h	0.939	0.973	0.979	0.968	0.288	0.297	0.982	0.984	0.981	−0.138	0.139
9 km/h	0.966	0.955	0.962	0.947	0.756	0.921	0.965	0.965	0.960	0.642	0.781
10 km/h	0.979	0.967	0.977	0.961	0.630	0.654	0.978	0.983	0.976	0.194	0.330
MEAN	0.935	0.972	0.976	0.968	0.392	0.155	0.979	0.982	0.978	0.022	0.044

**Table 4:** ICC results for energy expenditure computed from the ActiGraph sensor on two placements (thigh and wrist).

Speed	Crombach’s Standardized Items	Energy Expenditure Calculation System									
		Freedson et al. 1998 [32]	Crouter et al. 2010 [30]	Santos-Lozano et al. VT 2013 [20]	Santos-Lozano et al. VM 2013 [20]	Sasaki et al. 2011 [33]	Corrected Freedson et al. 1998 [32]	Corrected Crouter et al. 2010 [30]	Corrected Santos-Lozano et al. VT 2013 [20]	Corrected Santos-Lozano et al. VM 2013 [20]	Corrected Sasaki et al. 2011 [33]
1.4 km/h	0.853	−0.352	−0.760	0.331	0.802	0.437	−0.468	−0.675	0.051	0.673	0.455
2.9 km/h	0.961	−0.143	−0.057	0.259	0.819	0.749	0.042	0.098	0.245	0.822	0.771
4.3 km/h	0.944	−0.237	−0.064	0.076	0.785	0.649	−0.107	0.230	0.066	0.690	0.654
5 km/h	0.948	−0.018	−0.022	0.185	0.529	0.473	0.173	0.272	0.284	0.549	0.632
6 km/h	0.946	0.050	0.068	0.187	0.404	0.484	0.194	0.232	0.275	0.612	0.710
6.5 km/h	0.755	−0.368	−0.420	−0.037	0.053	0.344	−0.212	−0.217	−00.30	0.257	0.521
7 km/h	0.923	−0.293	−0.296	−0.232	0.819	0.843	−0.330	−0.292	−0.332	0.709	0.796
8 km/h	0.957	0.243	0.227	0.144	0.508	0.542	0.501	0.368	0.447	0.279	0.419
9 km/h	0.929	0.034	0.056	−0.119	−0.073	−0.008	0.339	0.230	0.280	−0.257	−0.099
10 km/h	0.922	0.243	0.251	0.198	−0.585	−0.533	0.384	0.321	0.315	−0.583	−0.496
MEAN	0.921	−0.084	−0.102	0.099	0.406	0.398	0.052	0.057	0.133	0.375	0.436

## Data Availability

The datasets for this study can be found in: https://doi.org/10.6084/m9.figshare.12080730 (accessed on 30 April 2021).
